# Supplementary figures and images for: Updated range distribution of the non-native Asian green mussel Perna viridis (Linnaeus, 1758) at Guanabara Bay, Rio de Janeiro, Brazil
Source: PeerJ. 2024 Dec 19;12:e18649. doi: 10.7717/peerj.18649 (PMC11663401; doi:10.7717/peerj.18649)

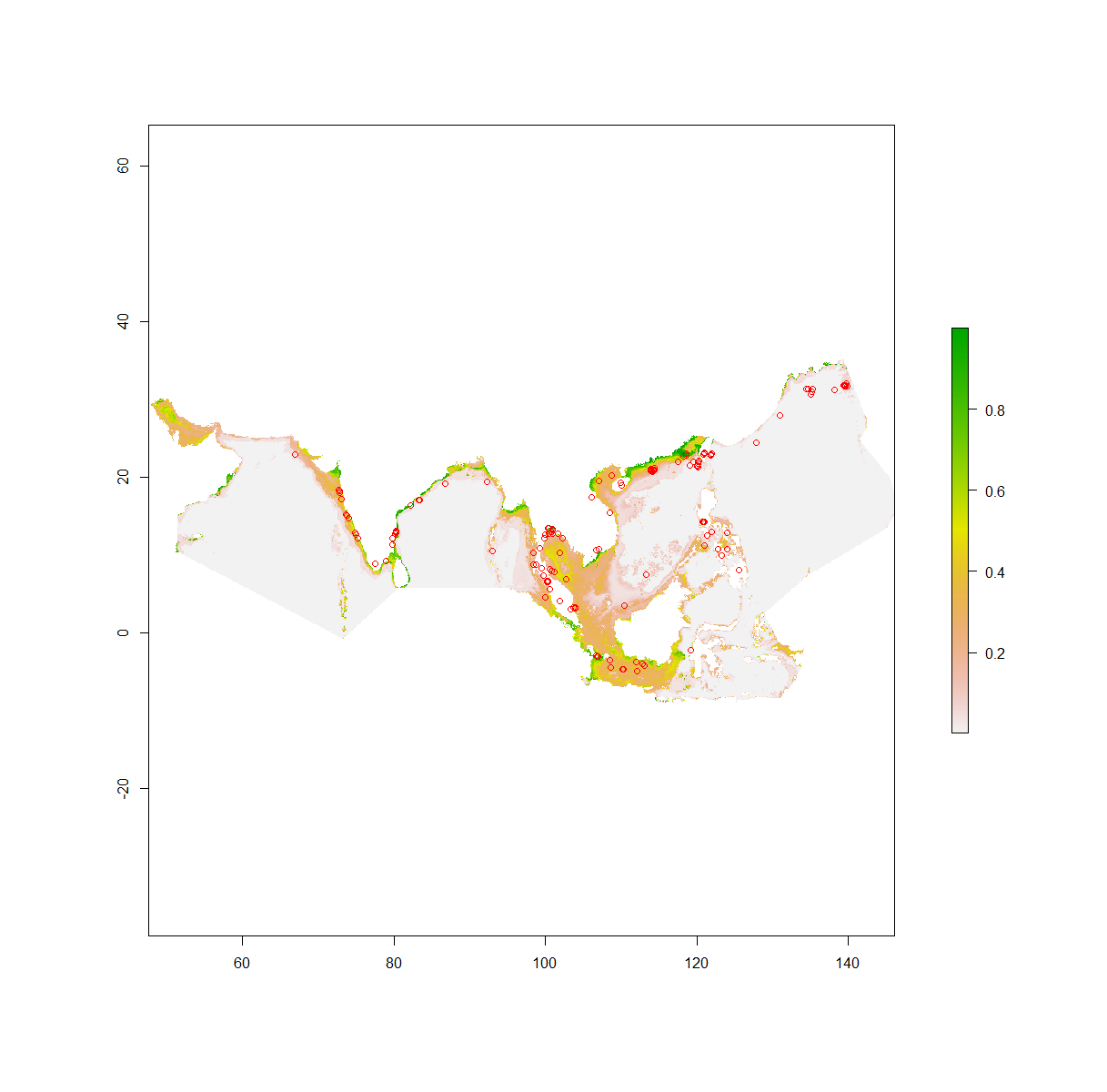

Supplement: Supplemental Information 3 [file peerj-12-18649-s003.png]
